# Factors affecting the oral health of inpatients with advanced cancer in palliative care

**DOI:** 10.1007/s00520-021-06547-5

**Published:** 2021-09-16

**Authors:** Junichi Furuya, Hiroyuki Suzuki, Rena Hidaka, Nei Koshitani, Yuko Motomatsu, Yuji Kabasawa, Haruka Tohara, Yuji Sato, Shunsuke Minakuchi, Satoshi Miyake

**Affiliations:** 1 Department of Geriatric Dentistry, Showa University School of Dentistry, Tokyo, Japan; 2 Dysphagia Rehabilitation, Department of Gerontology and Gerodontology, Graduate School of Medical and Dental Sciences, Tokyo Medical and Dental University, Tokyo, Japan; 3 Gerodontology and Oral Rehabilitation, Department of Gerontology and Gerodontology, Graduate School of Medical and Dental Sciences, Tokyo Medical and Dental University, 1-5-45 Yushima, Bunkyo-ku, Tokyo, 113-8549 Japan; 4 Department of Oral Health Sciences for Community Welfare, Graduate School of Medical and Dental Sciences, Tokyo Medical and Dental University, Tokyo, Japan; 5 Department of Nursing, Tokyo Medical and Dental University Medical Hospital, Tokyo, Japan; 6 Department of Oral Care for Systemic Health Support, Graduate School of Medical and Dental Sciences, Tokyo Medical and Dental University, Tokyo, Japan; 7 Center for Innovative Cancer Treatment, Tokyo Medical and Dental University Medical Hospital, Tokyo, Japan

**Keywords:** Cancer, Dysphagia, Oral health, Palliative care, Terminal care

## Abstract

**Purpose:**

Patients with terminal cancer undergoing multidisciplinary palliative care often have oral health problems, but these details are still unclear. This cross-sectional study aimed to elucidate the oral health of patients with terminal-stage cancer who are inpatient recipients of acute-phase palliative care, and to unveil the factors affecting their oral health.

**Methods:**

Participants were 121 patients with terminal-stage cancer (68 males, 53 females, mean age: 73.6 ± 11.1 years) and oral health complaints. They received palliative care at Tokyo Medical and Dental University Medical Hospital between April 2017 and August 2019. Their demographic and medical details were extracted, retrospectively, from their medical records, and their oral health status, such as the number of natural teeth, removable denture usage, Oral Health Assessment Tool (OHAT), and Dysphagia Severity Scale, were evaluated. All outcomes were assessed by a dentist from the palliative care team.

**Results:**

The problems with soft tissue, saliva, and oral cleanliness were observed. The absence of posterior occlusal support was common, and the use of removable dentures was often inadequate. In contrast, swallowing function was relatively well-conserved and 46.3% of the participants were capable of nutrition intake solely by mouth. Multiple regression analysis revealed a significant association between total OHAT score and age, consciousness level, prognostic level, and method of nutritional intake.

**Conclusion:**

The results revealed that the oral health of terminal cancer patients under palliative care declined despite receiving routine oral care from nurses, and suggest the importance of including dental professionals in multidisciplinary palliative care.

## Introduction

Patients with terminal-stage cancer often receive palliative care through multidisciplinary collaboration that aims to prevent and alleviate distress through early and accurate evaluation and management of pain along with other physical, psychosocial, and spiritual problems [1]. Early introduction of palliative care can also improve quality of life (QOL) and minimize discomfort during the final stages of life [2, 3]. As in many parts of the world, cancer is the leading cause of death in Japan, responsible for approximately 370,000 deaths in 2019 [4]. As a result, improving palliative care for patients with cancer has received significant focus. In this backdrop, since palliative care was included under Japan’s national health insurance in 1990, palliative care wards and care teams have been widespread in medical facilities of Japan [5].

Debilitation, fatigue, anorexia, pain, and depression are common in patients with terminal-stage cancer [6]. However, alongside systemic symptoms, patients also often develop various oral health disorders such as dry mouth, candidiasis, dysphagia, mucositis, dysgeusia, ulcers, dental caries, gingivitis, and tongue plaque [7]. These oral health problems are highly likely to have a major detrimental effect on QOL, both functionally, in terms of mastication, conversation, and eating difficulty, and psychologically, in terms of anxiety and depression [8]. Oral health management by a dental professional has been found to not only alleviate oral health problems, but also improve QOL by increasing the enjoyment of eating and improving communication through conversation [9]. Consequently, the active participation of dentists and dental hygienists in the multidisciplinary collaborative practice of palliative care, to improve oral health through proper management of oral hygiene and function, is extremely important for maintaining and improving the QOL of patients with terminal-stage cancer [10]. Nevertheless, there is little collaboration between palliative care teams and dental professionals [11], and palliative care teams are unaware of the findings related to oral health management in patients with terminal-stage cancer [12]. As a result, there is often limited participation of dental professionals in palliative care. A national survey of internists and nurses engaged in palliative care in Japan explored the need for dental intervention and revealed that despite almost all respondents recognizing the need for dental intervention, only limited dental intervention occurred [13].

To provide a scientific rationale for the active involvement of dental professionals in interdisciplinary palliative care and for the provision of proper oral management, the oral health of patients with terminal-stage cancer needs to be understood; however, the evidence in the literature is inadequate. Therefore, we performed a cross-sectional study to elucidate the oral health of older adult patients with terminal-stage cancer receiving acute-phase inpatient palliative care. Furthermore, the study aimed to reveal the factors with a detrimental effect on the oral health of patients with terminal-stage cancer.

## Methods

### Participants

The inclusion criteria for participants were the following: (1) patients had terminal-stage cancer; (2) there were complaints of oral health problems by patients, their family, or nurses; and (3) patients received palliative care at Tokyo Medical and Dental University Medical Hospital between April 2017 and August 2019. Complete data were available for 121 patients (68 males, 53 females). Written information was used to explain the study to all participants, including that the study would use anonymized medical information. Informed consent was obtained with an opt-out policy before the study could begin. The study was approved by the Tokyo Medical and Dental University Faculty of Dentistry Institutional Review Board (approval number: D2016-077).

### Outcomes

Information on participants’ age, sex, clinical outcome, systemic diseases, activities of daily living (ADL), consciousness level, days to death from the first dental examination (“days to transfer” for transferred participants), and method of nutritional intake was extracted from medical records, retrospectively. Oral outcomes were assessed at bedside during the first dental examination during palliative care. At the time of the oral assessment, the participants were receiving routine oral care from nurses, but not professional oral intervention from dental professionals. All outcomes were assessed by a dentist, who was one of the members of the Tokyo Medical and Dental University Medical Hospital palliative care team.

Clinical outcomes were assessed as death or discharge from the hospital. Prognostic levels were scored on a 4-point scale according to the days to death from the first round of dental examination (0–7 days, 0; 8–28 days, 1; 29–56 days, 2; 57 days or more and/or survival to discharge, 3). This classification was chosen because previous studies have reported that the terminal stage of cancer is generally 1–2 months [14] and that ADL rapidly declines 2–3 weeks before death [15].

The presence of systemic diseases was scored using the Charlson Comorbidity Index (CCI) [16]. The CCI utilizes a total score by rating the diseases as follows: coronary artery disease, congestive heart failure, chronic pulmonary disease, gastric ulcer, peripheral vascular disease, mild liver disease, cerebral vascular disease, connective tissue disease, diabetes mellitus, and dementia as 1; hemiplegia, kidney disease, diabetes mellitus with organ damage, malignancy diagnosed within 5 years, leukemia, and lymphoma as 2; moderate to severe liver disease as 3; and metastatic solid tumor and acquired immunodeficiency syndrome (AIDS) as 6. Moreover, we evaluated the site of primary cancer.

Consciousness level was determined based on the Japan Coma Scale (JCS) [17] that ascertained whether the patient was lucid (0), awake (I), roused by stimulation (II), or could not be roused by stimulation (III) [17].

ADL was scored on a 5-point scale based on performance status (PS) [18], where an individual who was “fully active and able to carry on all pre-disease ADL without restriction” was rated 0, “restricted in physically strenuous activity but ambulatory and able to carry out work of a light or sedentary nature” was rated 1, “ambulatory and capable of all self-care but unable to carry out any work activities; up and about more than 50% of waking hours” was rated 2, “capable of only limited self-care; confined to bed or chair more than 50% of waking hours” was rated 3, and “completely disabled; cannot carry on any self-care; totally confined to bed or chair” was rated 4.

The oral outcomes extracted were current number of teeth and of functional teeth, status of occlusal support related to masticatory performance and the method of nutritional intake [19, 20] according to the Eichner classification [21], removable denture usage, overall assessment of oral health according to the Oral Health Assessment Tool (OHAT) [22], whether specialist oral management is required according to a dental professional, and swallowing function according to the Dysphagia Severity Scale (DSS) [23]. The current number of teeth was the number of remaining natural teeth; the number of functional teeth was the number of teeth including dentures, implants, and other prosthetic teeth. Tooth stumps were excluded from both these criteria. The status of occlusal support was classified into three grades according to the Eichner classification, which is based on existing occlusal contact between the maxilla and mandible teeth in the premolar and molar regions. Therefore, the status of occlusal support was classified as follows: participants who had four occlusal support zones (A); participants who had one to three zones of contact or contact in the anterior region only (B); and participants who had no occlusal contact at all, although a few teeth could still remain (C). Removable denture usage was determined based on four categories: not required, wearing, owned but not worn, and not owned. The need for removable dentures was determined based on the occlusal support provided by the remaining natural teeth and overall health status; in particular, removable dentures were judged “required” if all posterior occlusal support was absent on one or both sides, if eating was impaired by the loss of teeth, and if consciousness level was rated in single digits according to the JCS. Participants who required removable dentures and wore them were categorized as “wearing.” Participants who had removable dentures but were unable to wear them due to a denture failure or incompatibility were categorized as “owned but not worn.” Participants who did not own removable dentures were classified as “not owned.” Finally, those judged not to require removable dentures were categorized as “not required.”

OHAT is used to assess oral health and scores the lips, tongue, gums and mucosa, saliva, natural teeth, dentures, oral cleanliness, and dental pain on a 3-point scale ranging from 0 to 2, with higher total scores indicating poorer overall oral health. A dentist assessed whether specialist oral management was needed.

The DSS is a global assessment of dysphagia severity rated on a 7-point scale, where lower scores indicate a greater severity of dysphagia. The DSS assessment was performed by a dentist based on the physician’s opinion. The method of nutritional intake [24] was scored on a 4-point scale: intravenous nutrition only (0), tube feeding (combined enteral and intravenous nutrition, or enteral nutrition only) (1), combined tube feeding and oral nutrition (2), and oral nutrition only (3).

### Statistical analysis

Clinical statistical analysis was performed on participant characteristics, oral outcomes, and methods of nutritional intake. Moreover, to determine which factors from among systemic diseases, prognosis level, and method of nutritional intake affected total OHAT score, a multiple regression analysis was performed using the forced entry method with total OHAT score as the dependent variable and age, sex (male: 0, female: 1), CCI, JCS score, prognostic level based on the first round of dental examination, method of nutritional intake, and aspiration (yes/no) based on DSS score (DSS 1–4, aspiration present [0]; DSS 5–7, no aspiration [1]) as independent variables. PS was subsequently removed as an independent variable because of its strong correlation with JCS. Moreover, as the current number of teeth, number of functional teeth, and state of occlusal support were already addressed by the OHAT comprehensive assessment of oral health, these outcomes were also removed as independent variables. Analysis was performed using SPSS Ver.26 (IBM Japan), and a significance level of 0.05 was used for all statistical analyses.

## Results

### Participant characteristics

Participant characteristics are shown in Table 1. The mean age and standard deviation of the participants was 73.6 ± 11.1 years. Almost all participants were older adults, with 14 participants aged ≤ 60 years (11.6%), 42 aged 61–74 years (34.7%), and 65 aged ≥ 75 years (53.7%). Head and neck cancers, known to result in a high incidence of oral complications such as dry mouth [25], and lung cancer were common among the study participants, although a wide range of primary cancer sites were also observed, including bladder and kidney cancers. The consciousness level was relatively good in 87.6% of participants, although 75.2% experienced some form of ADL limitation. Moreover, 85.1% of the participants died as inpatients, of whom 62% died within a month after the first dental examination.

**Table 1:** Characteristics of inpatients receiving palliative care for terminal-stage cancer (*N* = 121)

Variable	Category	Mean ± SD	Median	*n*	%
Age (years)		73.6 ± 11.1	75	121	
Sex	Male			68	56.2
	Female			53	43.8
Outcome	Death			103	85.1
	Discharge			18	14.9
Days to death based on first dental examination		28.4 ± 30.0	20	121	
Prognostic level based on first dental examination	0–7 days			22	18.2
	8–28 days			53	43.8
	29–56 days			30	24.8
	≥ 57 days			16	13.2
CCI		4.9 ± 2.0	6	121	
Primary cancer site	Lung cancer			23	19.0
	Head and neck cancer			22	18.2
	Bladder cancer			8	6.6
	Colorectal cancer, small Intestine cancer			8	6.6
	Pancreatic cancer			8	6.6
	Kidney cancer			8	6.6
	Liver cancer			7	5.8
	Prostate cancer			6	5.0
	Gastric cancer			5	4.1
	Esophageal cancer			5	4.1
	Malignant lymphoma			5	4.1
	Other			16	13.3
JCS	0			54	44.6
	I			52	43.0
	II			14	11.6
	III			1	0.8
PS	0			0	0.0
	1			8	6.6
	2			22	18.2
	3			37	30.6
	4			54	44.6

*CCI* Charlson Comorbidity Index, *JCS* Japan Coma Scale, *PS* Performance Status

### Oral health, need for oral management, and method of nutritional intake

**Fig. 1:**
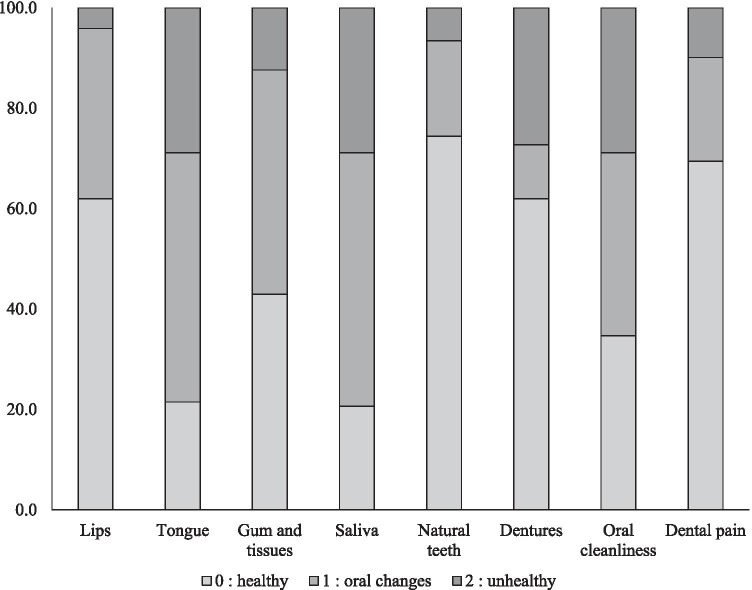
OHAT category scores in terminal-stage cancer patients receiving palliative care

Results related to oral health, need for oral management, and methods of nutritional intake are shown in Table 2. Participants had a relatively large number of remaining natural teeth, although 24.8% were placed in the Eichner classification “group C” due to a complete loss of occlusal support from natural teeth. No removable dentures were needed by 46.3% participants; of the remaining participants who required removable denture, 64.6% did not use it. For all participants, the mean total OHAT score was 5.6 ± 2.8, and the median score was 5, indicating poor oral health. Observing OHAT results by category (Figure 1) revealed problems related to soft tissue categories (tongue, gums, and mucosa), as well as saliva and oral cleanliness categories in at least 50% of the participants. Overall assessment by a dentist showed that 65.3% of the participants required specialist dental intervention. The median DSS score, which rates swallowing ability, was 5 (no aspiration but oral problems), showing that swallowing ability was relatively well conserved. Assessing the method of nutritional intake revealed that 46.3% of the participants were capable of intake solely by mouth, and when participants with combined tube feeding and oral intake were included, 62% of them were capable of some form of nutritional intake by mouth.

**Table 2:** Oral health, need for specialist oral management, and method of nutritional intake of inpatients receiving palliative care for terminal-stage cancer (*N* = 121)

Variable	Category	Mean ± SD	Median	*n*	%
Current number of teeth		18.9 ± 9.9	22	121	
Number of functional teeth		22.3 ± 8.0	26	121	
Eichner classification	A			45	37.2
	B			46	38.0
	C			30	24.8
Removable denture use	Not owned			24	19.8
	Owned but not worn			18	14.9
	Wearing			23	19.0
	Not required			56	46.3
DSS score		4.2 ± 1.8	5	121	
	1			12	9.9
	2			20	16.5
	3			5	4.1
	4			23	19.0
	5			21	17.4
	6			32	26.5
	7			8	6.6
OHAT	Total score	5.6 ± 2.8	5	121	
	Lips	0.4 ± 0.6	0		
	Tongue	1.1 ± 0.7	1		
	Gums and mucosa	0.7 ± 0.7	1		
	Saliva	1.1 ± 0.7	1		
	Natural teeth	0.3 ± 0.6	0		
	Denture	0.7 ± 0.9	0		
	Oral cleanliness	0.9 ± 0.8	1		
	Dental pain	0.4 ± 0.7	0		
Need for specialist oral management	Not needed			42	34.7
	Needed			79	65.3
Method of nutritional intake	Intravenous only			34	28.1
	Tube feeding			12	9.9
	Combined oral and tube feeding			19	15.7
	Oral only			56	46.3

*DSS* Dysphagia Severity Scale, *OHAT* Oral Health Assessment Tool

### Factors affecting OHAT score

The results of the multiple regression analysis are shown in Table 3. A significant association was found between total OHAT score and age, consciousness level, prognostic level, and method of nutritional intake.

**Table 3:** Multiple regression analysis with total OHAT score as the dependent variable (*N* = 121)

Independent variables	*B*	SE	95% CI	*β*	*P* value	*VIF*
Age	− 0.049	0.021	− 0.090 to − 0.009	− 0.196	0.018*	1.137
Sex	0.032	0.449	− 0.856 to 0.921	0.006	0.943	1.080
CCI	− 0.051	0.113	− 0.276 to 0.173	− 0.037	0.653	1.126
JCS	1.084	0.402	0.288 to 1.880	0.273	0.008*	1.747
Prognostic level based on first round of dental examination	− 0.83	0.245	− 1.315 to − 0.344	− 0.274	0.001*	1.114
Aspiration	− 0.23	0.546	− 1.312 to 0.852	− 0.041	0.675	1.626
Method of nutritional intake	− 0.466	0.119	− 0.861 to − 0.071	− 0.214	0.021*	1.428

Multiple *R* = 0.579; *R*^2^ = 0.335; *P* < 0.001

*B* partial regression coefficient, *SE* standard error, *CI* confidence interval, *VIF* variance inflation factor, *β* standardized partial regression coefficient; Sex: male = 0, female = 1. Prognostic level based on dental round examination: 0–7 days = 1, 8–28 = 2, 29–56 = 5, ≥57 = 4. Aspiration (yes/no): aspiration present = 0 (DSS: 1–4), no aspiration = 1 (DSS: 5–7). Method of nutritional intake: intravenous only = 0, tube feeding = 1, combined tube and oral = 2, oral only = 3

All other independent variables were used as continuous variables. *CCI* Charlson Comorbidity Index, *JCS* Japan Coma Scale. * *P* < .05, Multiple regression analysis

## Discussion

This study revealed that almost all palliative care inpatients at Tokyo Medical and Dental University Medical Hospital, an acute care hospital, were older adult patients with a relatively good consciousness level. However, many participants experienced limited ADL. Participants also had poor oral health. In particular, problems related to soft tissue (tongue, gum, and mucosa), saliva, and oral cleanliness, which are common among patients with terminal-stage cancer, were observed [6, 7, 10]. Therefore, many patients required specialist dental interventions, such as professional oral care by dental hygienists including scaling and tooth brushing instructions, and denture repair and caries treatment by dentists. In contrast, swallowing ability was relatively well conserved in participants, and more than half of them were capable of nutritional intake through the mouth. The results also suggested that, even after adjusting for age and gender, oral health tended to be lower in patients with a decreased level of consciousness, shorter prognosis, and those who did not take oral nutrition.

The mean total OHAT score of the study participants was 5. A previous study that assessed the oral health of inpatients at an acute care hospital for stroke [26] and a study that assessed the oral health of patients receiving care from a nutrition support team at an acute care hospital [27] reported a median total OHAT score of 4. This suggests that the current study participants, who received palliative care in an acute care hospital, had poor oral health compared to inpatients of acute care hospitals in general. In particular, the percentage of participants in this study with problems related to soft tissues, such as tongue and gingiva, dry mouth, and mouth cleanliness problems, was high at 50% or more. This is consistent with previous reports of poor oral health characteristics in patients with terminal-stage cancer [6, 7, 10]. Therefore, the decline in the OHAT score in this study may be due to the effects of cancer progression and treatment. Tsai et al. [6] reported that, in patients with terminal-stage cancer, dry mouth and other oral health problems did not improve over time with palliative care. However, it is worth noting that dental professionals did not participate in Tsai et al.’s [6] study. Another report also found that oral health improved with the participation of dental professionals in multidisciplinary collaborative care during the acute phase [26]. In line with those findings, the results of this study support the need for active participation of dental professionals and for specialist oral health management in palliative care. In particular, treatment by dental specialists is likely to improve dry mouth, oral cleanliness, and oral mucosal involvement. Problems related to removable dentures were also common among the participants. Removable dentures are often removed in hospitals because of the complexities of denture management, dangers of aspiration and accidental ingestion, and impaired patient sensation. In this study, more than half of the participants did not wear their removable dentures despite needing them. Wearing removable dentures improves the ability to masticate and improves QOL by allowing patients to eat a variety of foods [28]. The practice is extremely important for establishing oral nutrition due to its effect on food transport when swallowing [29] and the support it provides for oral and pharyngeal movement during oral nutrition [20]. Although swallowing ability was relatively well-conserved in most participants of this study and at least 60% were capable of some form of intake by mouth, wearing removable dentures properly could further improve QOL by allowing even more patients to consume a less modified diet by mouth. As the compatibilities and incompatibilities of removable dentures can only be addressed by dentists, the above findings suggest that dental professionals have a key role in palliative care in terms of supporting nutritional intake via dentures.

Matsuo et al. [30] divided patients receiving palliative care into two groups based on days to death and compared the oral health between the two groups; the shorter prognosis group had a higher incidence of oral health problems such as glossitis and dry mouth and a higher proportion of patients with dysphagia who required oral care. Ohno et al. [31] reported poorer oral health in inpatients receiving non-oral nutrition than in those receiving oral nutrition and noted that, in particular, speech, swallowing, saliva, and the tongue were significantly worse. Previous reports and the present study revealed that prognostic level, in terms of days to death and nutritional intake status, had a negative effect on oral health. This suggests that if specialist dental intervention, such as oral care, is provided as part of palliative care, it must take into consideration the prognostic level and nutritional intake status of the patient.

This study has several limitations. First, it had selection bias because it included only patients with terminal-stage cancer and oral problem complaints by a patient, family, or nurse. In addition to cancer, patients with heart failure, dementia, and various other life-threatening diseases are recipients of palliative care [32]. Hence, the current findings on oral health may not apply to all patients in palliative care. However, this is not the only study showing the importance of oral care for patients with terminal cancer [33], and therefore, the active participation of dental professionals in the multidisciplinary collaborative practice of palliative care is extremely important. Second, this research was a cross-sectional study conducted at a single site; therefore, oral health is not causally related to the level of consciousness, prognosis, or method of nutritional intake. A longitudinal study is needed to reveal the effects of collaborative treatment provided by a dental health-oriented professional multidisciplinary team on the oral health of patients receiving palliative care, and the effects of this collaborative treatment on nutritional intake status, prognostic level, and patient QOL. However, considering the reports that dental intervention was an important care in palliative care to relieve patients’ suffering and maintain their quality of life [34], and that nutritional support tailored to the wishes of patients and their families was effective in improving the quality of life of palliative care patients [35], this study is clinically important because it suggests that many patients receiving palliative care in an acute care hospital need specialist dental intervention to improve their oral health, such as by oral hygiene management or prosthodontic treatment, and the active participation of dental professionals in the multidisciplinary collaborative practice of palliative care is important in improving the oral health of patients in a manner that gives due consideration to their general health status, estimated prognosis, and method of nutritional intake.

In conclusion, the study finding—that patients with terminal cancer receiving palliative care had poorer oral health related to consciousness level, prognostic level, and method of nutritional intake, despite receiving routine oral care by nurses—suggests the usefulness of dental professionals participating in the multidisciplinary collaborative practice of palliative care.

## Data Availability

Not applicable.
